# Comparison of the Results of Patients Treated With Limb-Sparing Treatment Options in Malignant Bone Tumors: Sharing the Experience of Twelve Bicentric Patients

**DOI:** 10.7759/cureus.46270

**Published:** 2023-09-30

**Authors:** Abdulsamet Emet, Yunus Demirtas, Ataberk Beydemir, Mehmet Ayvaz

**Affiliations:** 1 Orthopedics and Traumatology, Etlik City Hospital, Ankara, TUR; 2 Orthopedics and Traumatology, Yuksek Ihtisas University Medical School, Ankara, TUR; 3 Orthopedics and Traumatology, Hacettepe University, Ankara, TUR

**Keywords:** tumor, liquid nitrogen, bone sarcoma, extracorporeal irradiation, biological reconstruction

## Abstract

Objectives: Extremity-sparing surgery should be the main objective if a functional extremity is to be obtained in cases of malignant bone tumors. After extensive resection, numerous techniques have been described to reconstruct bone defects. This study aimed to compare the outcomes of patients who underwent external radiation therapy and liquid nitrogen biological reconstruction at two different facilities.

Methods: The study included 12 patients who received biological reconstruction therapy for bone sarcoma and had at least two years of follow-up. Demographic data, pathological diagnosis, presence of systemic metastasis, and recurrence during follow-up were among the information logged. Patients who used liquid nitrogen were placed in group 1, and those who underwent external irradiation were placed in group 2. After being contacted for their final follow-up appointments, the outcomes were compared by recording the Musculoskeletal Tumor Society Score (MSTSS).

Results: For participants with a mean age of 10.75±3.6 (5-17), the follow-up period was 30.2±16.3 months in total. In contrast to the patients in group 1, who experienced union on average after 7.5±1.2 months, those in group 2 experienced union after 7.6±1.1 months. Patients in group 1 had an MSTSS of 75.5±11.8%, while those in group 2 had a score of 77±4.4. There was no discernible difference between the two groups' union times (p>0.05). There was no statistically significant difference between the two groups' MSTSS (p>0.05).

Conclusion: After tumor resection, extracorporeal radiation therapy and the application of liquid nitrogen are still useful treatment options and neither of them is superior to the other.

## Introduction

Bone and soft tissue sarcomas make up 1% to 10% of all malignant tumors [1]. The survival rates for bone and soft tissue sarcomas have significantly increased in recent years as a result of the development of multidisciplinary treatment approaches [2]. At this point, neoadjuvant treatment techniques used particularly during the preoperative period, surgical techniques used afterward, and adjuvant treatments have all proven successful. Surgical practices played a significant role in this multidisciplinary approach. Wide surgical margins are always necessary for these surgical applications [3,4].

Reconstruction of bone defects that develop after extensive resection of malignant tumors is a special issue in limb-preserving treatment. The surgical procedure that will be used to fill these defects needs to be durable and low risk of complications. There are two techniques that can be applied to the treatment for this reason. Endoprosthetic replacement is one of them, and biological reconstruction is the other. Bone defects are reconstructed effectively with endoprosthetic reconstructions. In addition to benefits such as good functional outcomes and postoperative weight-bearing, there are drawbacks such as prosthesis infection, loosening, and high cost. Endoprosthetic reconstructions are, therefore, rarely used in the pediatric age range [5,6].

Biological reconstructions are another technique for treating bone defects caused by tumors. The use of the patient's bone stock, whether the bone is active or inactive, is necessary for this purpose. The techniques used in reconstructions with living bone are osteocallotasis and segment shifting or vascularized bone grafts [7]. Although technically challenging, these surgical procedures are effective methods with high rates of union, low costs, and long-term survival because viable bone tissue is present. The techniques for bone reconstruction with inactive bone tissue include massive allografts and reclamation. However, large-scale allografts and endoprosthetic reconstructions are expensive and have limited application because of risks such as infection and immune reaction.

Regenerated bones have benefits such as being less expensive and being the patient's own graft. It is essentially possible to recycle using two different approaches. The first of these involves removing the tumor tissue from the body and fixing it after external irradiation sterilizes it [8]. The second involves fixing the tissue following liquid nitrogen treatment after the tissue has been removed from the body [9]. Both of these methods are being used more frequently, and the outcomes are acceptable. This study aimed to compare the outcomes of patients who underwent external radiation therapy and liquid nitrogen biological reconstruction at two different facilities.

## Materials and methods

Following the local ethics committee's approval (Hacettepe University, 2022/19-28), 16 patients with bone sarcoma who underwent biological reconstruction using two distinct techniques in two different centers between 2017 and 2022 were included in the study. The study excluded a total of four patients whose records could not be located. According to the Enneking classification method, data on age, sex, side, localization, pathological diagnosis, systemic metastasis and recurrence in the follow-up, infection in the follow-up, treatment type, and tumor stage at the time of diagnosis were collected. Group 1 included patients who recovered using liquid nitrogen, while group 2 included patients who recovered using external irradiation. The Musculoskeletal Tumor Society Score (MSTSS) was recorded, the patients were contacted for their last control, and the outcomes were compared.

After imaging techniques for the tumor were used on each patient, the diagnosis of the tumor was verified histopathologically with a true-cut biopsy. By using computed tomography (CT) and positron emission tomography (PET) systemic scans of the lung and abdomen, all patients with histopathologically confirmed diagnoses were staged in accordance with the Enneking staging system. Additionally, for preoperative and surgical planning purposes, two-way radiography and contrast-enhanced magnetic resonance imaging (MRI) were performed on the lesion. Following surgical planning, the tumor was removed using either surgical technique, leaving a 2 cm surgical margin and the biopsy tract within the incision.

**Figure 1:**
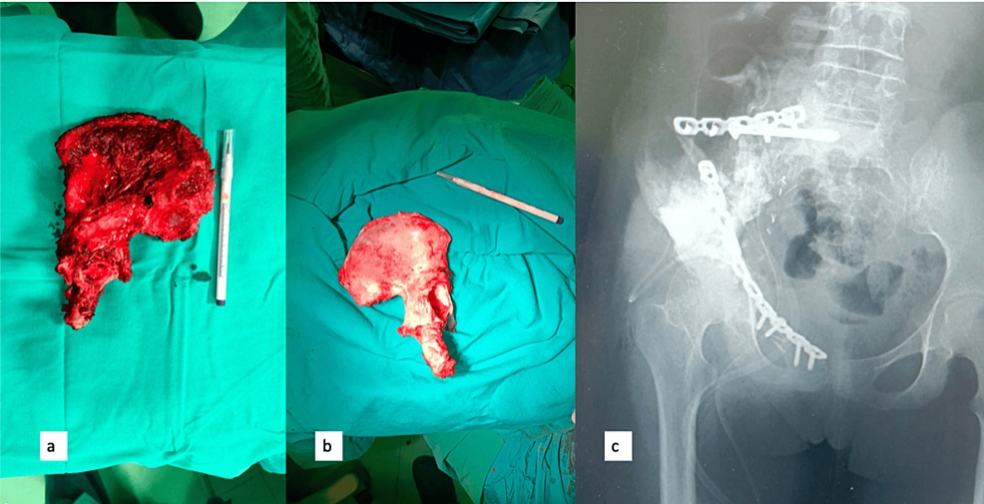
Clinical pictures of Ewing sarcoma patient using liquid nitrogen and biologic reconstruction; (a) clinical picture after tumor removal; (b) the prepared graft after the bone is cleared from tumor and liquid nitrogen treatment; (c) post-operative x-ray

**Figure 2:**
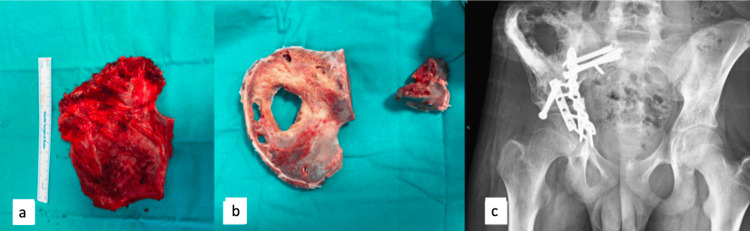
Clinical pictures of Ewing sarcoma patient with extracorporeal irradiation treatment; (a) clinical picture after tumor removal; (b) the prepared graft after bone is cleared from tumor and extracorporeal irradiation treatment; (c) post-operative x-ray

After the tumor was removed from patients undergoing surgery with liquid nitrogen, the soft tissues were cleaned. The intramedullary region tissues were curetted and sent for pathology. After being exposed to liquid nitrogen for 20 minutes, the autograft was then incubated at room temperature for 15 minutes. The prepared autograft was then applied to the damaged area, and internal fixation was used after it had been kept in a physiological saline solution at room temperature for 10 minutes (Figure 1). After tumor excision with a 2 cm surgical margin, the soft tissues and intramedullary area were cleaned and sent to pathology in patients who underwent surgery using the external irradiation technique. After that, it was covered with two layers of sterile drape containing 2 g of vancomycin and a sterile nylon drape before being transported to the radiotherapy unit. Internal fixation was carried out by applying the autograft to the defective area after external irradiation with 50 Gy for 25 minutes. Grafts were swiftly brought to the operating room in sterile conditions without deterioration. The prepared autograft was then applied to the resection area, and internal fixation was used (Figure 2).

All patients received adjuvant chemotherapy the third week after their wounds healed. Direct radiography and MRI were used to check the patients for local and systemic recurrence at three-month intervals for the first two years and six-month intervals after the second year. All patients began partial weight-bearing at three months. All patients were followed up with full weight-bearing after six months. According to the presence of callus tissue in at least three of the total four cortices in the control direct radiographs, the patients’ bones were considered as union. For their final follow-ups, all patients were contacted, and MSTSS was recorded.

Statistical analysis was in the Statistical Package for the Social Sciences (SPSS) 22 package program. Descriptive statistics results were in medians (minimum-maximum) and frequencies with percentages. The analyses were the Mann‒Whitney U-test, chi-square test, and Fisher’s exact test. The results had a 95% confidence interval, and the statistical significance was at the p<0.05 level.

## Results

Eight of the 12 study participants were men, and four were women. The mean age was 10.75±3.6 (5-17). One patient in group 1 had surgery for osteosarcoma, and the other 11 patients had Ewing sarcoma, according to an examination of the patients' pathological diagnoses. Four of the patients in group 1 had the iliac wing, the proximal femur in one, the distal femur in one, and the proximal tibia in the other when the affected areas in these patients were examined. In group 2, the affected areas included the iliac wing in one patient, the femoral shaft in two patients, the distal femur in one patient, and the tibial diaphysis in one patient. One patient received a classification of 3b under the Enneking classification, while all other patients received a classification of 2b. A total of 30.2±16.3 months were spent on follow-up. The mean follow-up time for patients in group 1 was 33.2±20.3 months, whereas it was 25.8±7.9 months for patients in group 2. The MSTSS of patients in group 1 was 75.5±11.8%, while that of patients in group 2 was 77±4.4%. There was no statistically significant difference between the two groups' MSTSS (p>0.05) (Table 1 and Table 2).

**Table 1:** Patients and demographic data NO, nitric oxide

Case	Age	Gender	Location	Localization	Surgery Type	Pathology
1	5	M	Left	Proximal femur	Resection + NO + Plate Fixation	Ewing
2	9	M	Right	Iliac wing	Internal Hemipelvectomy + NO + Plate Fixation	Ewing
3	7	M	Left	Femur, distal	Resection + NO + Plate Fixation	Osteosarcoma
4	7	M	Right	Iliac wing	Internal Hemipelvectomy + NO + Plate Fixation	Ewing
5	13	M	Right	Iliac wing	Internal Hemipelvectomy + Cementoplasty + NO + Plate Fixation	Ewing
6	13	F	Right	Iliac wing	Internal Hemipelvectomy + NO + plate fixation	Ewing
7	18	F	Left	Proximal tibia	Resection + NO + Plate Fixation	Ewing
8	11	M	Left	Femur, distal	Resection + External Irradiation + Plate Screw	Ewing
9	10	F	Right	Femoral shaft	Resection + External Irradiation + Plate Screw	Ewing
10	10	F	Right	Femur, distal	Resection + External Irradiation + Plate Screw	Ewing
11	15	M	Right	Iliac wing	Resection + External Irradiation + Plate Screw	Ewing
12	11	M	Left	Left femoral shaft	Resection + External Irradiation + Plate Screw	Ewing

**Table 2:** Bone union times and MSTSS of patients MSTS: Musculoskeletal Tumor Society Score

	Case	Metastasis	Recurrence	Union 6th Month	Resection (cm)	Follow-up Duration (mo)	MSTSS (%)	MSTSS p-value	Follow-up Duration p-value	Union Time p-value
Group 1	1	Lung metastasis	Negative	9	22	70	83	0.805	0.632	0.969
	2	Negative	Negative	7	18	27	86			
	3	Negative	Negative	8	20	39	50			
	4	Negative	Negative	6	19	24	78			
	5	Negative	Negative	8	19	23	76			
	6	Bone and bone marrow metastasis	Negative	6	21	25	80			
	7	Negative	Negative	9	14	25	76			
Group 2	8	Negative	Negative	8	13	21	73			
	9	Negative	Negative	7	25	34	80			
	10	Negative	Negative	8	15	25	83			
	11	Negative	Negative	6	17	24	76			
	12	Negative	Negative	9	14	25	73			

All patients were monitored by evaluating the lesion area with contrast-enhanced MRI and chest CT for systemic metastasis at three-month intervals for the first two years and at six-month intervals after the second year.

One of the patients who underwent liquid nitrogen and plate screw fixation due to conventional osteosarcoma of the distal femur experienced recurrence at 12 months. After a second operation, the patient received endoprosthetic reconstruction. Due to a distal femur Ewing sarcoma, a patient who underwent plate screw osteosynthesis and external radiation developed a superficial infection. The patient's cultures were taken, but there was no growth there. After receiving antibiotic therapy, he was taken for a debridement procedure as his discharge continued. Because there was no discharge in the fourth week following the operation, adjuvant therapy was initiated.

## Discussion

The most important finding of the study is that liquid nitrogen and external irradiation are both safe and effective treatment methods for biological reconstruction. There was no difference between the union times and MSTSS of the two groups. The results of both methods are not significantly different from each other.

The treatment of bone defects that will develop during the surgical removal of a malignant bone tumor is crucial if an extremity-sparing surgical procedure is to be used. Endoprosthesis reconstructions used to treat defects are more effective in the short to medium term but suffer in the long term due to factors such as infection, loosening, and cost [4,10]. The 10-year survival rate following endoprosthetic replacement has been found to range from 50% to 76% in the literature [9,11]. Allografts have drawbacks, including the potential for disease transmission, the requirement for a graft bank, ease of accessibility, and immunological reactions, even though they are used in biological treatment [8]. Methods of biological reconstruction are, therefore, vital for saving lives. Biological reconstruction techniques that are frequently used include pasteurization, liquid nitrogen sterilization, external irradiation, and allografts. Additionally, segment shifting and vascularized fibula grafts are technically challenging, but there is no disputing their superiority in biology. The benefits of autografts generated with external irradiation and liquid nitrogen include no disease transmission, anatomical compatibility, ease of reimplantation of ligaments and tendons, low cost, and bone stock preservation [12-14]. Additionally, this graft retains its osteoinduction and osteoconduction properties, which are crucial aspects of reacquisition [15]. It is also free of tumors. These biological treatment options offer excellent long-term survival and financial value. Regarding the outcomes of patients who were treated with liquid nitrogen and external irradiation, there is little information in the literature.

Gage et al. first used liquid nitrogen in 1966 to study how it affected bone tissue, which led to the development of sterilization from tumor tissue. After treating the mandible bone with liquid nitrogen in 1976, Marciani et al. demonstrated good remodeling of reimplantation [16,17]. With the advancement of surgical methods, liquid nitrogen can now be used in limb-sparing procedures. In their study from 2005, Tsuchiya et al. extensively described the method used to regenerate bone after tumor tissue has been removed. Only two of the 28 patients in their study reported poor outcomes, and 20 of them had excellent outcomes [14]. In a different study, liquid nitrogen was administered to a total of 10 patients, and after an average of 39.6 months, Garg et al. discovered that the MSTSS was 80% [18].

Restoration with external irradiation has been used in biological reconstruction for a very long time and was first used in 1968. Although when it was first written about in the literature, it was only used for bone defects, and it is now used for osteoarticular bone defects [19]. The technique's primary goal is to deliver high-dose radiation outside the body. After applying osteoarticular grafts to a total of 16 patients suffering from acetabulum tumors, Gundavda et al. looked at the long-term functional outcomes of recovery following external irradiation and discovered that the mean MSTSS was 87% after 24 months [19]. In a different study by Arpornchayanon et al., 24 out of 30 patients survived after 47 months, with a mean MSTSS of 87% [12]. In a total of 14 disease series, Xu et al. followed up for an average of 133 months; 10 patients survived at the end of this time, with a mean MSTSS of 25.34.7 [20].

Long-term follow-up of both techniques has some challenges. These include issues such as infection, graft resorption, delayed union, and nonunion. Garg et al. treated just one patient with a vascularized/nonvascularized fibular graft while performing 16 osteotomies on 10 patients, 15 of whom unionized in an average of 5.2 months. Infection, graft resorption, or recurrence were not found in any of the patients [18]. Another study that compared the outcomes of liquid nitrogen application versus external irradiation involved 164 patients in total. Of these, 79 received external irradiation treatment, while 84 received liquid nitrogen treatment. The patient's joint showed signs of nonunion. Recurrence was observed in 12 of the patients who received external radiation and in nine of the patients who received liquid nitrogen. Therefore, they did not discover any noteworthy difference between the two groups [21]. Yao et al. found nonunion in 27.28% of patients who received liquid nitrogen and 16.67% of patients who received external irradiation in a different study. Infection was seen in 1% of patients overall [4].

The most frequent complications, such as union problems, recurrence, infection, and fracture, were not observed in this study when both groups were assessed together. Only one of the seven patients to whom we applied liquid nitrogen had a recurrence in the first year. At one year, this patient's MSTSS was 50%. We discovered that the average MSTSS of the patients to whom we administered external irradiation in the first year was 76.16%. Both groups employed rigid fixation to ensure union, which is consistent with the literature, and to stop graft resorption. Weight-bearing and immobilization were delayed due to the long-term healing of the bones. This principle has been found to eliminate the risk of prolonged union or fracture when applied to patients.

There are still some limitations to this research. The study's brief follow-up period could have an impact on the findings. Long-term follow-up is still being performed in this regard. The limited patient population is another drawback. The outcomes might change if both patient groups were larger. Our study's retrospective nature and focus on the lower extremities is another drawback.

## Conclusions

In conclusion, liquid nitrogen and external irradiation are both secure and efficient therapeutic modalities in the biological reconstruction of bone sarcomas. The outcomes of the two methods do not significantly differ from one another. Their low price and ease of accessibility are also significant benefits. As the number of patients undergoing biological reconstruction using these methods increases and follow-up periods become longer, more light will be shed on this subject. It should also be considered that bone union should be examined in more detail in future studies.
